# First Evidence of *Leishmania infantum* Antibodies in Sheep (*Ovis aries*) from Southern Germany

**DOI:** 10.3390/ani14131860

**Published:** 2024-06-24

**Authors:** Benjamin Ulrich Bauer, María Eugenia Lebrero, Martin Ganter, Teresa Navarro, Antonio Fernández, Marta Ruíz de Arcaute, Aurora Ortín, Sergio Villanueva-Saz, Diana Marteles, Héctor Ruiz, María Climent, Pablo Quílez, Delia Lacasta

**Affiliations:** 1 Clinic for Swine and Small Ruminants, University of Veterinary Medicine Hannover, Foundation, Bischofsholer Damm 15, 30173 Hannover, Germany; benjamin.bauer@tiho-hannover.de (B.U.B.); martin.ganter@tiho-hannover.de (M.G.); 2 Animal Pathology Department, Veterinary Faculty, University of Zaragoza, Miguel Servet 177, 50013 Zaragoza, Spain; wlebrero@unizar.es (M.E.L.); teresanarr@gmail.com (T.N.); afmedica@unizar.es (A.F.); martarda@unizar.es (M.R.d.A.); aortin@unizar.es (A.O.); hectorruiz353@gmail.com (H.R.); pquilezloz1109@gmail.com (P.Q.); dlacasta@unizar.es (D.L.); 3 Clinical Immunology Laboratory, Veterinary Faculty, University of Zaragoza, Miguel Servet 177, 50013 Zaragoza, Spain; 4 Small Ruminant Clinical Service, Veterinary Faculty, University of Zaragoza, Miguel Servet 177, 50013 Zaragoza, Spain; mariacli@unizar.es; 5 Anatomy, Embryology and Animal Genetics, Veterinary Faculty, University of Zaragoza, Miguel Servet 177, 50013 Zaragoza, Spain

**Keywords:** *Leishmania infantum*, sandfly, serology, sheep, surveillance, goats, vector-borne diseases, zoonosis

## Abstract

**Simple Summary:**

In Europe, the protozoan parasite *Leishmania infantum* is transmitted by sandflies. A recent study found that sheep had antibodies against this parasite, triggering questions about the role of these animals in the life cycle. Therefore, blood samples from sheep and goats from two southern German states, Baden-Wuerttemberg (BW) and Bavaria (BAV), were tested for antibodies using a method called ELISA. Details, such as the species, sex and age, were recorded to assess any association between seropositivity and these animal characteristics. A total of seven sheep flocks from BW and seven from BAV were included, comprising 274 sheep and 10 goats in BW, and 277 sheep and 78 goats in BAV. In BW, four sheep from three flocks had antibodies, and in BAV, the same number of sheep tested positive but from four different flocks. Overall, 1.45% of sheep had antibodies against *L. infantum*, while all goats tested negative. No significant links were found between the presence of antibodies and the factors examined. Our study shows that sheep in areas not typically associated with *Leishmania* can still be exposed to it. Further research is needed to determine if sheep could help us identify new areas where sandflies live and where the disease could spread.

**Abstract:**

In Europe, *Leishmania infantum* is the most prevalent *Leishmania* species, and this protozoan is transmitted by phlebotomine sandflies. A recent publication has shown that sheep harbor *L. infantum* antibodies. This raises questions about the epidemiological role of small ruminants. Therefore, sera from small ruminants located in two southern German federal states, Baden-Wuerttemberg (BW) and Bavaria (BAV), were analyzed with an ELISA to determine the presence of *L. infantum* antibodies. The species, sex and age (gimmer vs. ewe) were recorded, and a univariate analysis was conducted to determine possible associations. In total, seven sheep flocks (274 sheep/10 goats) from BW and seven sheep flocks (277 sheep/78 goats) from BAV were examined. In BW, four sheep from three flocks tested positive for *L. infantum* antibodies. In BAV, the same number of positive sheep were detected but in four flocks. The total seropositivity rate in sheep was 1.45%. All goats tested negative. No significant association (*p* > 0.05) was detected between *Leishmania* seropositivity and the variables evaluated. Our study reveals the exposure of sheep to *L. infantum* in a non-endemic area. Further investigation is needed to determine whether sheep can be used as sentinels to identify new phlebotomine habitats and *Leishmania* risk areas.

## 1. Introduction

The protozoan parasites of the *Leishmania* species are transmitted by phlebotomine sandflies and cause leishmaniasis in humans and leishmaniosis in animals. The zoonotic vector-borne disease is endemic in tropical and subtropical climates, including the Mediterranean basin [1]. In Europe, *Leishmania infantum* is the most prevalent *Leishmania* species, and this protozoan is transmitted by different species of phlebotomies [2]. Humans infected with *L. infantum* may develop either visceral leishmaniasis, including fever and splenomegaly, or cutaneous leishmaniasis, characterized by skin ulcers [3]. Dogs are considered the main domestic reservoir, and clinical manifestations of canine leishmaniosis cause cutaneous lesions, ocular and general abnormalities [1,3]. Moreover, other carnivores, such as cats, could have a direct impact as urban reservoirs in cities related to the stray cat populations in endemic areas of *L. infantum* infection [4,5]. In Africa and Asia, the exposure of small ruminants to *Leishmania* species has been reported [6,7], but this is less the case in Europe [8]. However, recent publications from Spain showed that almost 10% of tested sheep harbored *L. infantum* antibodies, and a clinical case of leishmaniosis in a goat with generalized exfoliative dermatitis was described [9,10]. This raises a question about the epidemiological role of sheep and goats in the *Leishmania* life cycle and whether small ruminants can act as reservoir hosts. The impact of climate change on vector distribution and vector-borne disease incidence is evident [11]. Its influence on leishmaniosis distribution is shown in the effect of temperature on sandflies’ development, although final proof for this hypothesis is still pending [2]. The northward expansion of *L. infantum* is largely attributed to the movement of infected dogs, presenting a significant challenge in countries with limited or absent populations of sandfly vectors, such as Germany [1]. In 1999, the first plebotomine sandflies, *Phlebotomus mascittii*, were detected in the southwest of the German federal state Baden-Wuerttemberg. In the following years, more *P. mascittii* were identified in this region, which is known as Upper Rhine Valley and has the hottest summer climate in Germany [12,13,14]. It is assumed that *P. mascittii* might transmit *L. infantum*, but its vector competence is still not proven [2]. Autochthonous cases of human/canine/feline and equine leishmaniosis in Germany are reported as a rare event, and Germany is still classified as a non-endemic country [13]. Information on the exposure to *L. infantum* in German small ruminants is missing. Sheep and goats are kept very extensively and used for conservation grazing, having frequent access to the natural habitat of vectors, and can, therefore, be used as sentinel animals for the surveillance of vector-borne pathogens [15,16]. The occurrence of sandflies in Southwest Germany is likely underestimated, and global warming may expand their habitat, increasing the risk of sandfly-borne infections [12]. These issues reinforce the necessity of *Leishmania* surveillance in Germany and the investigation of potential new reservoir hosts.

The first objective of the present pilot study was to identify *L. infantum* antibodies in sheep and goats originating from a German region known for its presence of *P. mascittii*. The second objective was to include small ruminant flocks from a district where phlebotomines have not been discovered before in the investigations to detect *L. infantum* antibodies in a previously unknown area. Our generated information will help to estimate the distribution of sandflies and *L. infantum*, which is in the spirit of the One Health approach by using small ruminants as sentinel animals [16].

## 2. Materials and Methods

**Figure 1:**
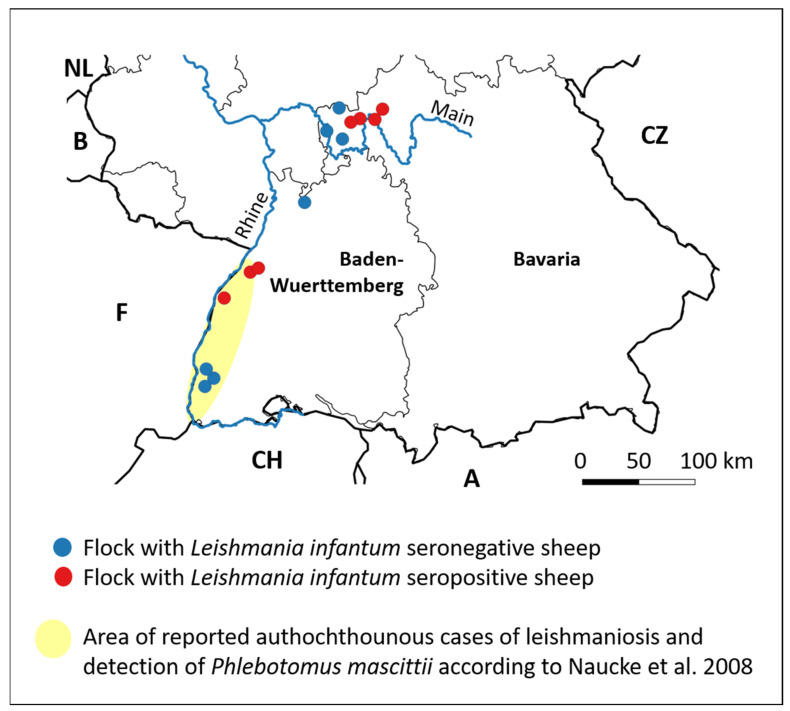
Geographical allocation of the 14 sheep flocks from southern Germany (Baden-Wuerttemberg and Bavaria), which were analyzed for *L. infantum* antibodies. Each point represents one flock.

Serum samples from small ruminants were obtained in Germany from a previous prevalence study on *Coxiella burnetii* antibodies [17]. The required sample size from each flock was calculated on the assumptions of a 3% expected prevalence, 95% confidence interval, 80% power and 5% precision, leading to a maximum of 44 animals sampled per herd. If goats were kept on the same farm, their sample size was independently calculated under the same assumptions, regardless of the number of sheep sampled. From this sample pool, sera from seven flocks (BW1–7) located in the federal state of Baden-Wuerttemberg were selected to determine *L. infantum* antibodies (Figure 1). These farms are situated in the Upper Rhine Valley. Additionally, seven sheep flocks (BAV1–7) located in the district Lower Franconia in the federal state of Bavaria were included in the study (Figure 1). In this area, phlebotomine vectors have not been previously detected, but it was predicted that this area would be suitable for sandfly species in the future due to climate change [18]. Most sheep farms were visited from the end of December 2017 until early March 2018, but farm BW5 was sampled in May 2018. Nine farms (BAV2–4, BW1–5, BW7) kept sheep, and five farms (BAV1, BAV5–7, BW6) consisted of sheep and goats. The species, sex and age of each animal were recorded. Based on their age and sex, the animals were classified as gimmers/doelings (=female nulliparous sheep/goat < 2 years old), ewes/does and rams/bucks (≥2 years old).

The enzyme-linked immunosorbent assay (ELISA) was performed on all sera, as described previously [10]. A 100 µL aliquot of sheep sera diluted 1:100 in PBST and 1% dry skimmed milk (PBST-M) was added to each well. The plates were incubated for 1 h at 37 °C in a moist chamber. After washing the plates for 3 min three times with PBST, followed by one wash with PBS for 1 min, 100 µL of Protein A/G conjugated to horseradish peroxidase (Thermo Fisher Scientific, Inc., Waltham, MA, USA) was added per well. Protein A/G conjugated is capable of interacting with the fragment crystallisable region from immunoglobulin G of all antibody species for several animals, including sheep, dogs, cats and ferrets. The plates were incubated for 1 h at 37 °C in the moist chamber and were washed again with PBST and PBS, as described above. The substrate solution (ortho-phenylene-diamine) and stable peroxide substrate buffer (Thermo Fisher Scientific, Inc., Waltham, MA, USA) were added at 100 µL per well and developed for 20 ± 5 min at room temperature in the dark. The reaction was stopped by adding 100 µL of 2.5 M H_2_SO_4_ to each well. Absorbance values were read at 492 nm in an automatic microELISA reader (Microplate Photometer Biosan Hipo MPP-96, Riga, Latvia). As a positive control, each plate included three serum samples, including a sick seropositive dog, a sick seropositive cat and a sick seropositive goat diagnosed with clinical leishmaniosis [9]. By contrast, sera from healthy, non-infected sheep were used as a negative control. The same positive and negative sera were used for all assays. All samples were run in duplicate. The cut-off in sheep was set to 0.38 optical density units (OD units) (mean + 3 standard deviations (SD) of values from 90 sheep from non-endemic areas such as northern Spain). In the case of goats, the cut-off was set to 0.26 OD units (mean + 3 standard deviations (SD) of values from 50 healthy goats from non-endemic areas such as northern Spain, mountainous area of the Basque Country and Huesca, areas where livestock farmers have not documented cases of canine leishmaniosis). In all cases, results above these values were considered positive.

Data collected for the entire population were analyzed using descriptive statistics. Univariate analysis of categorical data was performed to determine possible associations between *L. infantum* seropositivity and the following variables: age, sex and farm. The significance of this difference was assessed using Fisher’s exact test or Chi-square. A *p* ≤ 0.05 was considered significant. The SPSS v.22 software (SPSS Inc., Chicago, IL, USA) was used.

## 3. Results

In total, 274 sheep and 10 goats from Baden-Wuerttemberg and 277 sheep and 78 goats from Bavaria were analyzed. In Baden-Wuerttemberg, four sheep from three flocks (BW1, BW2, BW6) tested positive for *L. infantum* antibody. In Bavaria, the same number of positive sheep were detected, but in four flocks (BAV1, BAV2, BAV4, BAV6) (Table 1). Finally, the global seroprevalence of *L. infantum* infection in small ruminants, including sheep and goats, was 1.25% (95% confidence interval [CI] 0.64–2.45%). In the case of sheep, the global seroprevalence was 1.45% (95[CI] 0.74–2.84). All 88 goats tested negative. When considering the regions, the seroprevalence in sheep in Baden-Wuerttemberg was 1.46% (95[CI] 0.57–3.69), and 1.44% (95[CI] 0.56–3.65) in Bavaria. More details are presented in Table 1 and Table 2.

No significant association (*p* > 0.05) was detected between *Leishmania* seropositivity and the variables age, sex and farm.

Additionally, new cut-offs of the ELISA technique were determined based on optical density from seronegative results from the animals included in this study. For this purpose, the new cut-off in sheep was set to 0.27 optical density units (OD units) (mean + 3 standard deviations (SD) of values from the 543 seronegative sheep), whilst the new cut-off in goats was set to 0.20 OD units (mean + 3 standard deviations (SD) of values from 88 seronegative goats).

## 4. Discussion

The epidemiological role of small ruminants in endemic areas of *L. infantum* infection is unknown. However, an epidemiological study performed in an endemic area of canine leishmaniosis in Spain detected a seroprevalence of 9.27% in sheep, based on the ELISA technique [10]. In the present pilot study, the number of seropositive sheep was lower compared to the findings of that Spanish serological study. This difference is probably due to the fact that Germany is not considered an endemic country of leishmaniosis. In sheep and goats from Greece where *Leishmania* is also endemic, no seropositive small ruminants were detected [19]. However, that Greek study utilized a different in-house ELISA, unfortunately hampering comparison with our results.

In Germany, to date, several thousand dogs imported from *Leishmania*-endemic countries have tested positive for *L. infantum*, making it the most prevalent vector-borne pathogen in this cohort [13,20]. Autochthones cases of leishmaniosis have hardly been reported in humans, dogs, cats and horses in Germany [13]. In dogs, in the absence of competent vectors for *L. infantum* transmission, other transmission routes are implicated, including venereal, vertical and bite wounds [21,22]. The presence of seropositive sheep together with the detection and identification of *P. mascittii* in the Upper Rhine Valley (Baden-Wuerttemberg) in the same year [12] suggest an exposure to *L. infantum*. The vector competence of *P. mascittii* to transmit *L. infantum* is still not confirmed. Nevertheless, *L. infantum* DNA has been detected in *P. mascittii* in several countries, such as Austria, Italy and Serbia [23,24,25]. Many *Leishmania* spp. are adapted to specific sandfly species for transmission [25]. In contrast, it is assumed that *L. infantum* is transmitted by multiple vectors, which makes the vector competence of *P. mascittii* more likely [25]. Therefore, further research is needed to prove this hypothesis.

The clinical impact of *Leishmania* spp. infection in small ruminants seems to be minor. The first case of leishmaniosis was reported in a goat in Kenya, exhibiting both visceral and cutaneous lesions, and *Leishmania aethiopica* was suspected to be the causative agent [26]. The second case in a goat was recently reported in Spain, with the diseased animal showing skin lesions for several months and *L. infantum* antibodies being detected [9]. After medication with anti-*Leishmania* drugs, the goat recovered. Amastigote stages of *Leishmania* spp. were detected in a sheep with a swollen ear and the overlying skin thickened and encrusted [27]. This is, so far, the only reported case in sheep of cutaneous leishmaniosis. After experimental infection of sheep with *Leishmania donovani* and of goats with *Leishmania major*, clinical signs were hardly observed, and the inoculated pathogens were not recovered from skin aspirates after 28 days and 42 days post-infection, respectively [28,29]. Only one sheep of six developed *L. donovani* antibodies [28]. The authors concluded that small ruminants are unlikely to be reservoirs for the tested *Leishmania* species [28,29]. However, those studies are more than 25 years old, and modern standard methods like PCR were not utilized. Incorporating such methods may provide new insights into the replication ability of *Leishmania* species in small ruminants. Nevertheless, the most recent autochthonous case of cutaneous leishmaniosis in a cow in Switzerland caused by *Leishmania* sp. *siamensis* [30] confirms the clinical susceptibility of ruminants to *Leishmania*, although such cases are rare and might depend on the *Leishmania* species. 

An important aspect of seroepidemiological studies is the adaptation of the cut-off technique, depending on whether the study is performed in an endemic or non-endemic area of *L. infantum* infection. This fact has direct implications, with differences between sensitivity and specificity values depending on whether these diagnostic parameters are estimated in non-endemic or endemic areas [31]. The ELISA technique used in our study was used previously to detect the presence of seropositive animals in endemic areas [10], with higher optical density cut-offs (0.38 in sheep and 0.26 in goats) in comparison with the cut-offs (0.27 in sheep and 0.20 in goats) described in the present study.

The presence of sheep with *Leishmania* antibodies shows the possible exposition to *L. infantum* in a traditionally non-endemic country. Moreover, the detection of four seropositive sheep in Southwest Baden-Wuerttemberg is a further indication of the existence of phlebotomies sandflies in the Upper Rhine Valley, as reported by others [12,13]. As a result of climate change, it is important to perform active and passive surveillance on vectors and vector-borne pathogens to obtain detailed information related to the potential spread to new geographical areas or the introduction of new pathogens in areas that were classified as non-endemic. In the present pilot study, four antibody-positive sheep were identified in Bavaria (Lower Franconia), an area where no phlebotomine sandflies were detected previously but where predicted favorable climate conditions for sandflies exist [18]. The three positive females were raised on the Bavarian sheep farms, but the origin of the seropositive ram cannot be identified anymore. Therefore, it is highly likely that at least the two ewes and the gimmer were exposed to *L. infantum* in Northwest Bavaria. To determine whether sheep can be used as sentinels to identify new sandfly habitats and *Leishmania* risk areas, light traps need to be set up in proximity to the sheep flocks with seropositive animals. This approach is compliant with the One Health concept, as leishmaniasis is also a serious disease in humans. The early detection of potential new risk areas will raise awareness for disease recognition and prevention, as autochthonous human leishmaniasis is still a rare disease in Germany [32]. However, it is predicted that factors such as climate change will trigger the further northward spread of *Leishmania* species, with the risk of new endemic countries such as Germany [2]. Moreover, potential clinical leishmaniosis has to be considered not only in small ruminants but also in herding dogs, which are frequently used for sheep flocks.

## 5. Conclusions

Our study reveals the exposure of sheep to *L. infantum* in a non-endemic country. Therefore, it is important to consider the possibility of clinical leishmaniosis in animals and humans in Germany, although it is still a rare event. Further investigations are needed to determine whether sheep can be used as sentinels to identify new phlebotomine habitats and *Leishmania* risk areas.

## Figures and Tables

**Table 1:** The numbers of sheep and goats according to their origin (German federal state) and sex analyzed for *Leishmania infantum* antibodies.

State	Female Sheep (Ewes/Gimmers)	Rams	Female Goats (Does/Doelings)	Bucks	Total (Sheep/Goats)
Baden-Wuerttemberg	259 (215/44)	15	10 (8/2)	0	274/10
Bavaria	247 (161/86)	30	73 (64/9)	5	277/78
Total	506 (376/130)	45	83 (72/11)	5	551/88

**Table 2:** Details of sheep tested positive for *Leishmania infantum* antibodies from Baden-Wuerttemberg and Bavaria, Germany.

State	Flock ID	Flock Seroprevalence (%) ([Confidence Interval])	Animal ID	Sex	Estimated Age (Year)	ELISA Result ^1^
Baden-Wuerttem-berg	BW1	4.76% [1.32–15.79]	556	Ewe	3	0.42
			1103	Ewe	3	0.39
	BW2	2.86% [0.51–14.53]	21277	Ewe	4	0.43
	BW6	1.92% [0.34–10.12]	0860	Ewe	7	0.40
Bavaria	BAV1	1.32% [0.23–7.08]	4259	Ewe	3	0.42
	BAV2	2.50% [0.44–12.88]	6902	Ewe	6	0.40
	BAV4	2.50% [0.44–12.88]	0362	Ram	4	0.41
	BAV6	2.38% [0.42–12.32]	15	Gimmer	1	0.47

^1^ ELISA positivity threshold: ≥0.38 optical density units (OD units).

## Data Availability

The datasets used and/or analyzed during the current study are available from the corresponding author on reasonable request.
